# Sequence-Based Screening for Rare Enzymes: New Insights into the World of AMDases Reveal a Conserved Motif and 58 Novel Enzymes Clustering in Eight Distinct Families

**DOI:** 10.3389/fmicb.2016.01332

**Published:** 2016-08-25

**Authors:** Janine Maimanakos, Jennifer Chow, Sarah K. Gaßmeyer, Simon Güllert, Florian Busch, Robert Kourist, Wolfgang R. Streit

**Affiliations:** ^1^Department of Microbiology and Biotechnology, Biocenter Klein Flottbek, University of HamburgHamburg, Germany; ^2^Junior Research Group for Microbial Biotechnology, Ruhr-University BochumBochum, Germany

**Keywords:** arylmalonate decarboxylase (AMDase), racemase, Variovorax sp., Polymorphum gilvum, decarboxylation, flurbiprofen, sequence-based screening, Hidden Markov Models

## Abstract

Arylmalonate Decarboxylases (AMDases, EC 4.1.1.76) are very rare and mostly underexplored enzymes. Currently only four known and biochemically characterized representatives exist. However, their ability to decarboxylate α-disubstituted malonic acid derivatives to optically pure products without cofactors makes them attractive and promising candidates for the use as biocatalysts in industrial processes. Until now, AMDases could not be separated from other members of the aspartate/glutamate racemase superfamily based on their gene sequences. Within this work, a search algorithm was developed that enables a reliable prediction of AMDase activity for potential candidates. Based on specific sequence patterns and screening methods 58 novel AMDase candidate genes could be identified in this work. Thereby, AMDases with the conserved sequence pattern of *Bordetella bronchiseptica*’s prototype appeared to be limited to the classes of *Alpha*-, *Beta*-, and *Gamma-proteobacteri*a. Amino acid homologies and comparison of gene surrounding sequences enabled the classification of eight enzyme clusters. Particularly striking is the accumulation of genes coding for different transporters of the tripartite tricarboxylate transporters family, TRAP transporters and ABC transporters as well as genes coding for mandelate racemases/muconate lactonizing enzymes that might be involved in substrate uptake or degradation of AMDase products. Further, three novel AMDases were characterized which showed a high enantiomeric excess (>99%) of the (*R*)-enantiomer of flurbiprofen. These are the recombinant AmdA and AmdV from *Variovorax* sp. strains HH01 and HH02, originated from soil, and AmdP from *Polymorphum gilvum* found by a data base search. Altogether our findings give new insights into the class of AMDases and reveal many previously unknown enzyme candidates with high potential for bioindustrial processes.

## Introduction

Arylmalonate decarboxylases (AMDase; EC 4.1.1.76) belong to the family of carboxy-lyases (EC 4.1) and are very rare and underexplored enzymes. AMDases are capable of decarboxylating a range of α-disubstituted malonic acid derivates to enantiopure products without the need for any cofactor. This cofactor independent and enantioselective decarboxylation could open the door to highly valuable, (enantio-) pure fine chemicals and important building blocks for pharmaceutical products with environmentally friendlier biotechnological methods rather than chemical processes. One example for these compounds are 2-arylpropionic acids which belong to the important class of non-steroidal, anti-inflammatory drugs (NSAID) like ibuprofen, flurbiprofen or naproxen (Kourist et al., 2011a). In the early nineties the patent for naproxen production expired and resulted in numerous investigations to optimize the production of the homochiral (*S*)*-*(+)-isomer (Harrington and Lodewijk, 1997), which is the almost exclusively pharmacologically active enantiomer (Harrison et al., 1970). The great interest in aryl alkanoic acids lead to the discovery of the first and best characterized AMDase from *Bordetella bronchiseptica* (Miyamoto and Ohta, 1992a) which was object of a large number of investigations. The crystal structure of the enzyme was solved and enabled a prediction of a putative reaction mechanism (Okrasa et al., 2008) that was recently confirmed by semi-empirical calculations (Lind and Himo, 2014). Furthermore, there were constant efforts to optimize this AMDase for biotechnological use. It started with the inversion of enantioselectivity from (*R*)*-*products to (*S*)-isomers (Ijima et al., 2005; Obata and Nakasako, 2010) and proceeded with enhancement of AMDase activity of mutant enzymes (Terao et al., 2006; Kourist et al., 2011a; Miyauchi et al., 2011; Yoshida et al., 2015; Gaßmeyer et al., 2016). Engineered variants of the AMDase are able to produce several chiral building blocks from readily accessible alkenyl malonates and a range of enantioenriched, heteroaromatic α-hydroxycarboxylic acids including pharmaceutical precursors (Okrasa et al., 2009; Lewin et al., 2015). The extensive knowledge about their structure and reaction mechanism makes them suitable candidates for further protein engineering approaches. But besides the intensive efforts of optimizing the enzymes’ catalytic capabilities for industrial application only little is known about their occurrence and putative metabolic function. AMDases are members of the widespread Asp/Glu racemase superfamily together with aspartate (EC 5.1.1.13) and glutamate racemases (EC 5.1.1.3), hydantoin racemases (EC 5.1.99.5) and maleate isomerases (EC 5.2.1.1). Modified variants of the AMDase from *B. bronchiseptica* showed racemase activity (Kourist et al., 2011b; Gaßmeyer et al., 2015). The mechanism of this promiscuous reaction is currently an active field of research (Busch et al., 2016). Nevertheless, there are only four enzymes with proven AMDase activity known until today (Miyamoto and Ohta, 1992a; Miyamoto et al., 2007, 2008; Okrasa et al., 2008) followed by a larger number of annotated ORFs which might encode further AMDases.

The aim of this work was to establish a sequence-based screening algorithm for a reliable prediction of enzymes with AMDase activity of *Bordetella*’s prototype, to classify newly discovered enzymes and to gain insights into the occurrence, spreading and genetic context of these biotechnologically promising enzymes.

## Materials and Methods

### Databases Used in This Study

Nucleotide and amino acid sequences of putative AMDases were acquired from the NCBI^1^ and IMG database (Integrated Microbial Genomes)^2^. Sequences were compared to others deposited in the NCBI database using BLAST alignment tools^3^ (Altschul et al., 1990). Structural information on the enzymes was retrieved from the RCSB-PDB database ^4^.

### Bioinformatic Analyses

Sequence data were processed using BioEdit^5^, Clone Manager Suite 9 (SciCentral software, licensed), Staden Package containing Pregap^6^ and Gap4 and SeaView^7^ 4.5.1/4.3.4 (Gouy et al., 2010). Maximum-likelihood phylogenetic trees based on amino acid sequences were constructed using SeaView^7^ 4.5.1 and 4.3.4 (Gouy et al., 2010)].

The representative amino acid sequence of the *B. bronchiseptica* KU1201 AMDase (IMG Locus Tag Ga0077226_10648), which included all known AMDase-specific motifs (Okrasa et al., 2008), was used for a protein blast search against the IMG and NCBI non-redundant database. Resulting hits with an *e*-value ≤ 1e-44 and an identity score ≥ 43% were analyzed and evaluated manually. In total, 63 candidate AMDases were identified, including 58 new enzymes.

Amino acid sequences of AMDases (**Supplementary Table S1**) were aligned using T-coffee 8.99 in sensitive mode. Subsequently, hmmbuild of the HMMER package 3.1b2 was used to construct a profile HMM (Hidden Markov Model) from the multiple alignment file. The HMM logo was visualized using the skylign webtool ^8^. The new domain will be retrievable from the Pfam database collection^9^.

### Cloning and Heterologous Expression of *amdA, amdV*, and *amdP* in *Escherichia coli* BL21

For strains and plasmids used in this study see **Tables 1–3**. Cloning of *amdA* (IMG locus tag Ga0099533_11097; 708 bp) and *amdV* (IMG locus tag Ga0099534_11097; 711 bp) into the expression vector pET21a (Novagen/Merck, Darmstadt, Germany) was accomplished after amplification from genomic DNA using gene specific primer pairs with underlined restriction sites (*amdA*_for: 5′-AGGGGATCCATGACGAAACCCCATCT-GGG-3′/*amdA*_rev: 5′-ATGAAGCTTGGCGAAAAGACGGCCGTGGC-3′ and *amdV*_*Nde*I_for: 5′-CATATGGTGACACAGCAGCCCCATC-3′/*amdV*_*Xho*I_rev: 5′-CTCGAGGGCGA-ACAGCAGTCCC-3′). The gene sequence of *amdP* (744 bp) was obtained from IMG (IMG locus tag SL003B_3169) and synthesized with codon usage adapted to *E. coli* (MWG Eurofins, Germany; sequence deposited in Supplementary Information). The constructs were verified by sequencing (MWG Eurofins, Germany) and were subsequently transformed into *E. coli* BL21. Cultures were grown aerobically in LB medium containing 100 μg/ml ampicillin at 37°C until they reached an OD_600_ of 0.5. After induction with 0.1 mM IPTG, the corresponding proteins were expressed at 22°C for 20 h harboring a C-terminal sixfold histidine tag. The enzymes AmdA, AmdP, and AmdV were purified from crude cell extract with nickel-ion affinity chromatography using Ni-NTA agarose (Qiagen, Hilden, Germany) and checked for the correct sizes by SDS-PAGE. The enzymes were dialyzed over night at 4°C against 10 mM EDTA (pH 7.5) in a SERVAPOR tube (SERVA, Heidelberg, Germany; Ø 16 mm, MWCO 12000-14000).

**Table 1:** Bacterial strains used in this work.

Strain	Properties^1^	Source
*Escherichia coli* DH5α	*supE*44 Δ*lacU*169 (Φ80 lac*Z*ΔM15) *hsdR*17 *recA*1 *endA*1 *gyrA*96 *thi*-1 *relA*1	Invitrogen [Karlsruhe, Germany; (Hanahan, 1983; Bethesda Research Laboratories, 1986)]
*Escherichia coli* EPI300	Host strain for fosmid libraries F^-^ *mcrA* Δ(*mrr-hsdRMS-mcrBC*) Φ80*dlacZ*ΔM15 Δ*lacX*74 *recA1 endA1 araD139* Δ(*ara, leu*) 7697 *galU galK* λ-*rpsL nupG*	Epicentre^®^ (Madison, WI, USA)
*Escherichia coli* BL21 (DE3)	F^-^, *ompT, hsdS* B (r_B_^-^ m_B_^-^) *gal, dcm*, λDE3	Novagen/Merck (Darmstadt, Germany)
*Achromobacter* sp. HH01	*amd*	Soil sample, Botanical Garden (Duisburg, Germany), enrichment culture with phenylmalonic acid as sole carbon source
*Variovorax paradoxus*	Type strain, DSM-30034	DSMZ (Braunschweig, Germany)
*Variovorax* sp. HH01	*amdA*	Soil sample, Botanical Garden (Duisburg, Germany), enrichment culture with phenylmalonic acid as sole carbon source
*Variovorax* sp. HH02	*amdV*	Soil sample, Botanical Garden (Duisburg, Germany), enrichment culture with phenylmalonic acid as sole carbon source

*^1^Geno- and phenotypes according to (Bachmann, 1983).*

**Table 2:** Vectors used in this work.

Vector	Properties^1^	Size (kb)	Source
pDrive	TA-cloning vector, *oriEc*, P_lac_*lacZ*, Amp^R^, Kan^R^, T7 promoter	3.85	QIAGEN (Hilden, Germany)
pET-21a	Expression vector, *lacI*, Amp^R^, T7-*lac-* promoter, C-terminal His_6_-tag coding sequence	5.44	Novagen/Merck (Darmstadt, Germany)
pCC1fos^TM^	Fosmid vector, Chl^R^, *red*F, *oriV, ori2, rep*E, *par*A, *par*B, *par*C, cos, *lox*P, *lacZα*, T7 Promoter	8.139	Epicentre^®^ (Madison, WI, USA)
pEXA	Cloning vector, Amp^R^, P_lac_*lacZ*, pUC *ori*	2.450	Eurofins MWG Operon (Ebersberg, Germany)

*^1^Geno- and phenotypes according to the manufacturer’s specifications.*

**Table 3:** Constructs created, resp. used in this work.

Construct	Vector	Insert size (kb)	Properties
pET-21a::*amdA*	pET-21a	0.708	*amdA* derived from *Variovorax* sp. HH01, inserted at *Bam*HI and *Hind*III restriction sites, with C-terminal His_6_-tag coding sequence, Amp^R^
pET-21a::a*mdV*	pET-21a	0.712	*amdV* derived from *Variovorax* sp. HH02, inserted at *XhoI* and *NdeI* restriction sites, with C-terminal His_6_-tag coding sequence, Amp^R^
pET-21a::*amdP*	pET-21a	0.745	*amdP* derived from *Polymorphum gilvum* SL003B-26A1, inserted at *XhoI* and *NdeI* restriction sites, with C-terminal His_6_-tag coding sequence, Amp^R^

### Enrichment Culture of Microorganisms from Soil Samples

Soil samples originated from the Botanical Garden in Duisburg, Germany, (latitude: N51°29′15.8″, longitude: E6°46′3.5″) and were incubated aerobically at 30°C in 100 mL flasks for 3 days with a selective enrichment medium [1 L contained 5.0 g NaNO_3_ × 3 H_2_O; 1.01 g K_2_HPO_4_ × 3 H_2_O; 0.25 g MgSO_4_ × 7 H_2_O; 0.015 g CaCl_2_ × 7 H_2_O; 0.14 g FeSO_4_ × 7 H_2_O; 0.02 g EDTA. All components were prepared as 100-fold concentrated solutions and autoclaved separately. The medium was supplemented with 10 mL of a trace element solution [100-fold containing 15 mg Co(NO_3_)_2_ × 6 H_2_O; 61.8 mg H_3_BO_4_; 198 mg MnCl_2_ × 4 H_2_O; 57.5 mg ZnSO_4_ × 7 H_2_O; 47.5 mg NiCl_2_ × H_2_O; 2.5 mg CuSO_4_ × 5 H_2_O; 2.4 mg Na_2_MoO_4_; 4 mg CaCl_2_ × 6 H_2_O) and 10 mM phenylmalonic acid (Sigma–Aldrich)]. For streak dilution, agar plates were prepared with the same medium and incubated at the same temperature to get single colonies, which were then transferred onto indicator medium agar plates [500 mL contained 0.5 g KH_2_PO_4_; 0.5 g yeast extract; 1 g NaCl; 1 g (NH_4_)SO_4_; pH was set to pH 6.0 with KOH/HCl. After autoclaving and cooling to 60°C, sterile filtered 0.025 g kresol red (solved in 70% ethanol) and 1.8 g phenylmalonic acid (solved in H_2_O, pH set to 6 with KOH) was added]. The low amount of yeast extract enabled a fast growth of cell mass. When yeast extract was depleted, phenylmalonic acid was the sole carbon source and its decarboxylation caused a pH increase. Colonies which showed a color shift after 2–3 days provided initial indication of using phenylmalonic acid as a carbon source and were further investigated.

### Biochemical characterization of AmdA, AmdP, and AmdV

For general activity tests, the three different AMDases were assayed using purified recombinant protein. Unless otherwise indicated, the enzymes were added to a substrate solution [10 mM phenylmalonic acid (Sigma–Aldrich) dissolved in 50 mM sodium phosphate buffer, pH 7.0]. The reaction was stopped by the addition of 2 M HCl and by putting the reaction tubes on ice. The decrease of substrate concentration was measured by isocratic, reverse-phase HPLC (LaChrom Elite^®^, VWR International GmbH, Germany) with RP-18 columns (LiChroCART 125-3, Puropsher RP-18E und LiChroCART 125-4, Purospher RP-18, VWR International GmbH, Germany). The elution solvent was composed of 80% sodium phosphate buffer (50 mM, pH 7) and 20% methanol with a flow rate of 0.5 ml/min. Analysis of measurements was performed by EZChrom Elite (version 3.2.1, licensed, VWR International GmbH, Germany). Retention times of phenylmalonic acid and phenylacetic acid were 1.5–2.5 min resp. 4.5–6.5 min, dependent on column diameter at a wavelength of 214 nm.

All samples were measured in triplicate with three biological replicates. For determination of the optimal temperature, samples were incubated between 4 and 50°C for 45 min. Temperature stabilities were assayed at 22 and 4°C for up to 30 h and at -20°C for up to 70 days. The impact of pH conditions on enzyme activity was measured using phenylmalonic acid substrate solutions prepared in 50 mM citric acid buffer (pH 4.0, 5.0 and 6.0), 50 mM sodium phosphate buffer (pH 6.0 and 7.0) or 50 mM potassium phosphate buffer (pH 6.0, 6.5, and 7.0) and 50 mM Tris buffer (pH 7.0, 8.0, and 9.0). The pH dependent enzyme activity of AmdA was measured using 20 mM phenyl malonic acid substrate solution solved in 100 mM citric acid buffer (pH 4.3, 5.2, 5.4, and 5.8), 200 mM potassium phosphate buffer (pH 5.8, 6.5, and 7.3) and 30 mM Tris buffer (pH 7.0 and 8.0).

The influence of salts and possible cofactors on AmdP, AmdV, and AmdA was assayed with the following compounds in 1 mM final concentration: sodium phosphate, pyridoxal phosphate, NAD, ATP, thiamine diphosphate, zinc chloride, sodium chloride, and magnesium chloride. The reaction using 10 mM phenylmalonic acid was stopped with 2 M HCl after 45 min.

To determine the solvent stability of the AMDases, ethanol, methanol, isopropanol and DMSO were added to the reaction mixture (10 mM phenylmalonic acid in 50 mM Tris-buffer, pH 7.0) to a final volume of 10% (v/v). The reaction was stopped after 45 min and the activity compared to solvent-free controls.

### Selectivity of AmdA, AmdV, and AmdP on Derivatives of 2-Arylpropionic Acids

**FIGURE 1:**
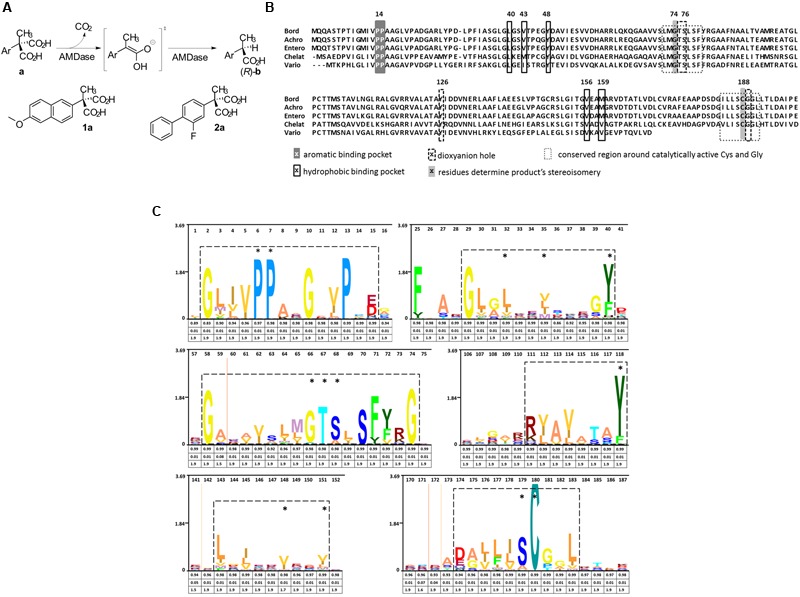
**Reaction mechanism and catalytic amino acids of AMDases. (A)** Reaction scheme showing the conversion of arylmalonic acids (a) to (*R*)-arylpropionic acids (b). Structural formula of the substrates naproxen (1a) and flurbiprofen (2a). **(B)** Amino acid residues involved in decarboxylation. Alignment of five verified AMDases showing the amino acid residues inolved in the catalytic process described by Okrasa et al. (2008). Gly74 and Cys188 determine stereoisomery of AMDase products, with Cys188 being the only proton donor. In racemases of this protein family, Gly74 is replaced by a second Cys which causes the production of racemates. Amino acids forming the dioxyanion hole set up stabilizing hydrogen bonds with the intermediate and product. Residues which are part of the hydrophobic binding pocket destabilize the removing carboxylic group while components of the aromatic binding pocket optimally position the aromatic group of AMDase‘s substrates. Bord; *Bordetella bronchiseptica* KU1201 (IMG locus tag Ga0077226_10648), Achro; *Achromobacter* sp. (tag not available), Entero; *Enterobacter cloacae* KU1313 (tag not available), Chelat; *Chelativorans* sp. BNC1 (IMG locus tag Meso_3050), Vario; *Variovorax* sp. HH01 (IMG locus tag Ga0099533_11097). **(C)** Hidden Markov Model (HMM) of AMDase amino acid motifs: An alignment of the AMDase amino acid sequences listed in **Supplementary Table S1** (Supplementary Information) was used to calculate a HMM profile. The HMM was consequently visualized as a logo (skylign.org). Six sequence motifs are framed which include all amino acids crucial for catalytic activity (marked with an asterisk) described by Okrasa et al. (2008). The information content (bits) is represented on the ordinate. The three values provided under each amino acid give information on the occupancy, insert probability and insert length, respectively.

The selectivity of the three different AMDases in the synthesis of optically pure 2-arylpropionic acids was analyzed by using purified recombinant protein. The enzymes were added to a substrate solution [10 mM **1a** or **1b** dissolved in 50 mM tris buffer, pH 7.5]. **1a** or **1b** (see **Figure 1A**) were prepared as described in (Gaßmeyer et al., 2016). The reaction was stopped by the addition of 2 M HCl and 2-arylpropionic acids were extracted twice with methyl *tert-*butyl ether (500 μL). Methanol (100 μL) and trimethylsilyl-diazomethane (25 μL) were added and the mixture incubated at room temperature for 30 min. Acetic acid (5 μL) was added, the solvents evaporated to dryness and the residue was re-dissolved in ethyl acetate (200 μL). Conversion and optical purity were then analyzed by chiral gas chromatography on a Shimadzu GC Plus 2010 device (Gaßmeyer et al., 2016); using a FS-Hydrodex-β-6TBDM column (Macherey-Nagel), with a method operating isothermic at 160°C (for naproxen) and 170°C (for flurbiprofen) with an injection split of 1/20. The elution order was for both 2-arylpropionic acids (*S*) and (*R*) with baseline separation and a peak difference of 0.5 min. *ee*-values were determined from the relative peak areas according to standard procedures. *ee* stands for enantiomeric excess and represents the excess of one enantiomer in a racemic mixture. Errors of *ee*-value determination were extrapolated from the area difference obtained from the respective racemic authentic standard. From this, the precision of *ee*-values was found to be ±0.3%.

## Results

### Conserved AMDase-Specific Amino Acid Motif as the Basis for a Search Algorithm

**FIGURE 2:**
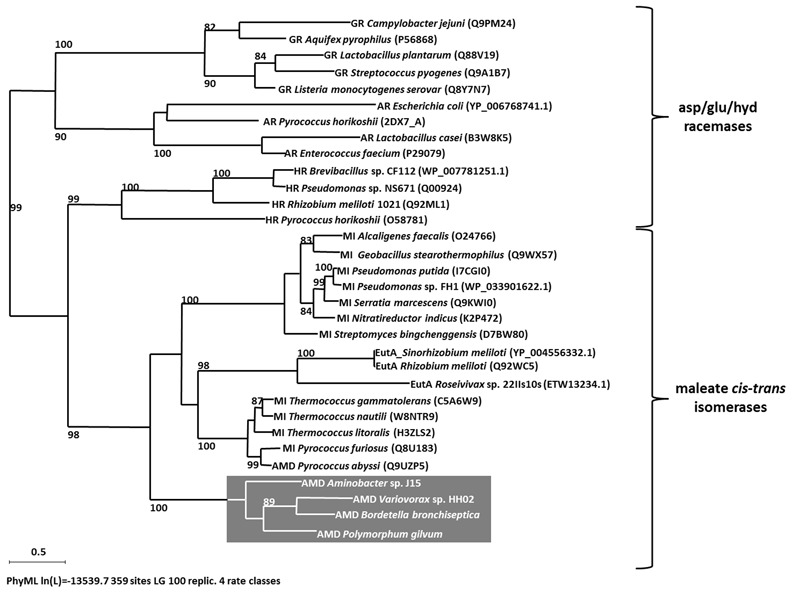
**Maximum likelihood tree (PhyML, LG) based on amino acid sequences of members of asp/glu racemases superfamily.** Bootstrap values ≥80% are mentioned. Scale bar corresponds to 0.5 substitutions per amino acid. Abbreviations: glutamate racemase (GR), aspartate racemase (AR), hydantoin racemase (HR), maleate *cis-trans* isomerase (MI), Ectoin utilizing enzyme (EutA), arylmalonate decarboxylases (AMD) are highlighted in gray.

For the determination of AMDase-specific searching criteria an amino acid sequence alignment of known AMDases was established initially (**Figure 1B**). We then verified, that the catalytically crucial amino acids described in the postulated reaction mechanism (Okrasa et al., 2008; Lind and Himo, 2014) are conserved within all representatives. For the alignment only enzymes were chosen which (i) have a proven AMDase activity of the order of *Bordetella’s* prototype and (ii) are able to decarboxylate phenylmalonic acid and more complex prochiral substrates. Five enzymes met these requirements (**Figure 1B**): Four of them are the characterized AMDases mentioned above and additionally one enzyme from *Variovorax* sp. HH01. The strain is an isolate of an enrichment culture derived from soil with phenylmalonic acid as sole carbon and energy source (see Material and Methods) and the respective enzyme was characterized in the course of this work. Based on the published AMDases and our *Variovorax* enzymes, we were able to establish a total of 12 conserved motifs that could be used to identify AMDase sequence homologs (**Table 4**). The conserved amino acids described in Okrasa et al. (2008) and listed in **Table 4** include all AMDase-specific protein structural motifs known today, like the hydrophobic pocket, the dioxyanion hole and the aromatic binding pocket and additionally two sequence motifs surrounding the residues, which determine the product’s stereoisomery. These conserved sequence motifs were used to screen data bases for further enzymes with AMDase activity. In these analyses only proteins, which met all 12 criteria with identical amino acids or amino acid residues with similar physicochemical properties were chosen. With the established search algorithm, 58 further ORFs which most likely encoded enzymes with AMDase activity were identified from the IMG and NCBI databases. The amino acid sequence alignment of these enzymes was used to create a Hidden Markov Model (HMM) shown in **Figure 1C** and enabled the deduction of six conserved sequence motifs (**Table 5**) which seemed specific for the prototype AMDase from *B. bronchiseptica* and include all known catalytically active residues. The HMM was implemented into the search algorithm and used for a reliable prediction of AMDase-active enzymes. AMDase are members of the protein superfamily aspartate/glutamate racemases, including aspartate racemases (EC 5.1.1.13), glutamate racemases (EC 5.1.1.3), hydantoin racemases (EC 5.1.99.5) and maleate *cis*-*trans* isomerases (5.2.1.1). AMDases of *B. bronchiseptica’s* prototype with their conserved sequence patterns are clearly defined within the superfamily, as can be seen in **Figure 2**. Characteristic catalytic elements are the dioxyanion hole, the hydrophobic pocket, the large binding pocket for aromatic residues and a thiolate hole for the accommodation of the deprotonated Cys residue. The search algorithm and deduced sequence patterns allow a well-defined distinction between “true” AMDases and other members of the aspartate/glutamate superfamily for the very first time.

**Table 4:** Detailed list of 12 determined searching criteria for the identification of AMDases in data bases (numbering of amino acid according to the AMDase from *Bordetella bronchiseptica* KU1201).

Number	Criteria	Function
(1)	P14 P15	Part of large binding pocket
(2)	T75 S76	Part of dioxyanion hole
(3)	Y126	
(4)	G189	
(5)	L40	Building blocks of hydrophobic binding pocket
(6)	V43	
(7)	Y48	
(8)	V156	
(9)	M159	
(10)	G74	Distinctive for AMDases instead of second active site Cys characteristic for the related racemases.
	C188	Protonation of postulated endiolat intermediate
(11)	LMGTSLSF	Conserved sequence motifs surrounding residues that determine product’s stereoisomery (positions 72–79 and 184–191).
(12)	ILLSCGGL	

**Table 5:** Conserved sequence patterns and catalytically active amino acids of AMDases and their function.

Number	Sequence pattern	Function of conserved amino acids
(1)	GLIV**PP**AxGxVPxE_(10-23)_	Binding of aromatic residues
(2)	G**L**GLxx**V**xxxG**Y**_(37-48)_	Destabilization of leaving carboxylic group
(3)	GAxxVxLM**GTS**LSFYRG_(66-82)_	G74 determines product’s stereoisomery; hydrogen bridge bond
(4)	RVAVxTA**Y**_(119-126)_	Hydrogen bridge bond
(5)	LxIxx**V**xx**V**_(151-159)_	Destabilization of leaving carboxylic group
(6)	DALLIS**CG**xL_(182-191)_	Active site Cys determines product’s stereoisomery, hydrogen bridge bond

*The letter x indicates an unconserved position within the sequence pattern. Numbering of amino acids is according to the AMDase from *B. bronchiseptica* KU1201. Catalytically active amino acids are printed in bold.*

### Classification of AMDases

**FIGURE 3:**
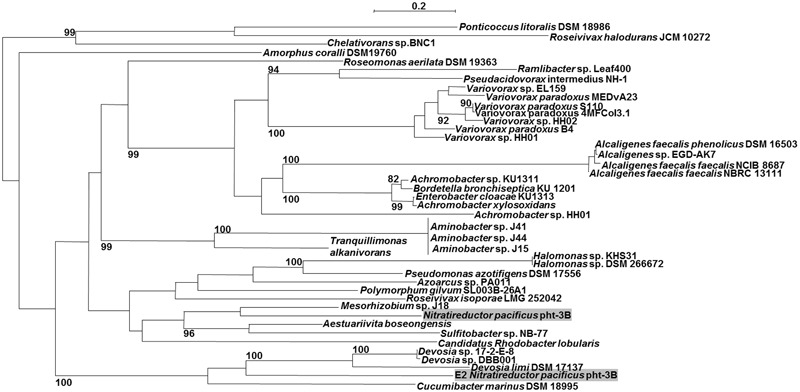
**Maximum likelihood tree (PhyML, LG) based on amino acid sequences of the AMDase genes.** Bootstrap values ≥80% are mentioned. Scale bar corresponds to 0.2 substitutions per amino acid. Both of *Nitratireductor‘s* enzymes are highlighted in gray. The tree was calculated with all known 63 enzymes, but for reason of clarity not all of them are shown.

**FIGURE 4:**
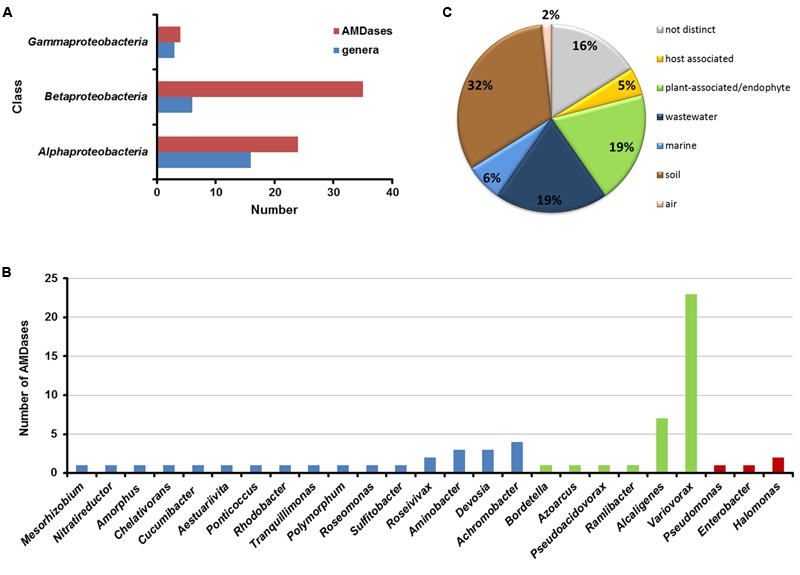
**Distribution of AMDase genes throughout the bacterial classes, genera and habitats. (A)** Number of AMDases compared to *amd*-carrying genera within proteobacterial classes. **(B)** *amd*-carrying genera within proteobacterial classes (blue: *Alpha-proteobacteria*, green: *Beta-proteobacteria*, red: *Gamma-proteobacteria).* **(C)** Habitats of AMDase-positive bacterial strains. The isolation sources of 63 AMDases have been evaluated (see **Supplementary Table S1** in Supplementary Information).

To date, 63 ORFs encoding putative AMDases were identified (status January 2016). All of the 25 *amd*-carrying bacterial genera belong exclusively to the class of *Alpha-, Beta-* and *Gamma-proteobacteria* (**Figure 3**). *Alpha-proteobacteria* include 25 *amd* genes, distributed over 16 genera, *Beta-proteobacteria* contain 34 AMDase genes distributed over six genera and within *Gamma-proteobacteria* four ORFs encoding AMDases were found, spread over three genera (**Figures 4A,B**). In strains with complete genome sequences, AMDase genes are encoded on the chromosomes. A singularity represents the strain *Nitratireductor pacificus* pht-3B which is the only isolate that encodes two different *amd* genes. *N. pacificus* pht-3B is part of a pyrene-degrading consortium of enriched sediment from the Pacific Ocean (Nie et al., 2012).

**FIGURE 5:**
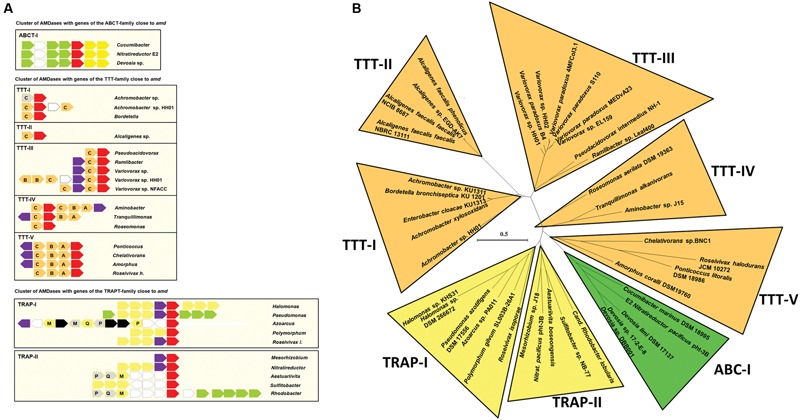
**Conserved homologous genes in the sequence environment of *amd* genes. (A)** Shown are genes encoding AMDases (red), mandelate racemase/muconate lactonizing enzymes (purple), TTT (orange), TRAP transporter (yellow), ABC transporter (green), and transposases (black). Gray arrows indicate genes that are annotated as hypothetical proteins but show similarities to genes in other strains. Unconserved genes are depicted as white arrows edged in gray. Letters denote subunits of the tripartite transporters. **(B)** AMDase clusters. Radial maximum likelihood tree (PhyML, LG) based on amino acid sequences of the AMDase-genes. Bootstrap values ≥80% are mentioned. Scale bar corresponds to 0.5 substitutions per amino acid. The tree was calculated with all known 63 enzymes, but for reason of clarity not all of them are shown.

It is intriguing that for most of the genera in which AMDases were identified; only one or two different strains with the *amd* genes could be detected. However, within the genus *Variovorax* 23 strains harbored an *amd* gene and within the genus *Alcaligenes* seven strains that carried *amd* genes were detected (**Figure 5B**). This observation may indicate that the *amd* gene was originally evolved within the genus *Variovorax* or a closely related group of bacteria. The findings also suggest that the *amd* gene is not part of an essential core genome of any of these bacteria but rather part of the accessory pan-genome.

Bacterial strains with *amd* genes derived from six isolation sources (**Figure 4C**): soil, plants (plant associated or endophytes), wastewater, oceans, hosts, and air. For 16% of the strains no distinct isolation source was mentioned. The majority (32%) is composed of strains that have been isolated from soil. All strains and gene locus tags are listed in **Supplementary Table S1** (Supplementary Information).

Comparison of *amd* flanking sequences enabled a more precise classification and characterization of the AMDases (**Figure 5**). One of the most widely spread ORFs was a gene encoding a mandelate racemase/muconate lactonizing enzyme (MR/MLE, e.g., IMG gene ID of *Variovorax* sp. HH01: 2639906160) which was present in 61% of the available sequences. Mandelate racemases (EC 5.1.2.2) and muconate cycloisomerases (EC 5.5.1.1) belong to the enolase superfamily and are involved in aromatic metabolism (Hasson et al., 1998). Moreover, genes commonly occurring were encoding tripartite tricarboxylate transporters (TTT). These sodium ion-dependent transporters translocate various substances, including citrate and its derivatives and are frequently found in *Beta-proteobacteria* (Antoine et al., 2003). Further genes cumulating in the sequence surrounding *amd* are ORFs coding for TRAP transporters and ABC transporters (Schneider and Hunke, 1998). TRAP transporters are tripartite ATP-independent periplasmic transporters which are common in prokaryotes (Mulligan et al., 2011). Like TTTs and ABC transporters, they consist of cytoplasmic membrane proteins and a periplasmic solute-binding protein.

Comparison of *amd* neighboring sequences combined with the amino acid sequence similarities allowed a first classification of all known AMDases into eight enzyme clusters (**Figure 5B**). Cluster TTT-I to TTT-V comprise isolates with genes encoding a TTT next to the *amd* genes. With exception of *Azoarcus* sp. all *Beta-proteobacteria* belong to the clusters TTT-I to TTT-III. Cluster TTT-IV and TTT-V are composed of *Alpha-proteobacteria*. By now there is only one cluster with *amd* genes flanked by ORFs encoding ABC transporters. This cluster includes the marine isolates *Cucumibacter marinus* DSM 18995 and *N. pacificus* pht-3B as well as the isolates of *Devosia* sp. There are two clusters of AMDases with ORFs encoding TRAP transporters nearby. Thereby the cluster TRAP-I is the only enzyme cluster with representatives of all three proteobacterial classes carrying AMDase coding genes.

### Biochemical Characterization of the Three Novel AMDases AmdA, AmdV, and AmdP

**FIGURE 6:**
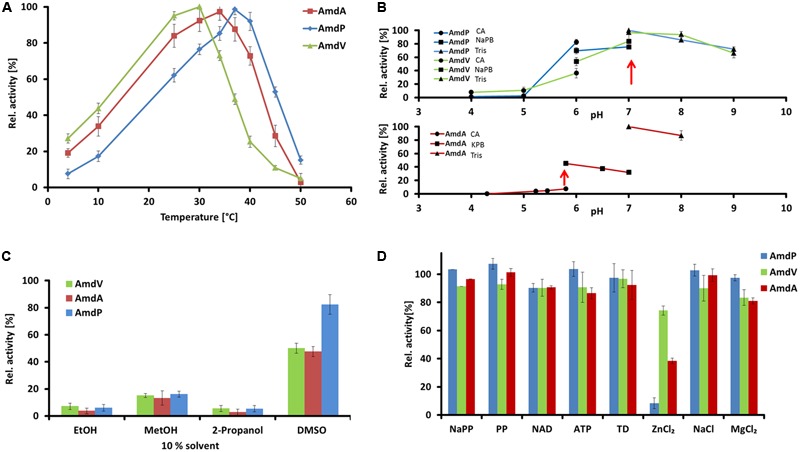
**Enzymatic properties of the three recombinant and purifed AMDases AmdA (*Variovorax* sp. HH01), AmdV (*Variovorax* sp. HH02), and AmdP (*Polymorphum gilvum* SL003B-26A1).** Measurements are triplicates with three technical repeats. **(A)** Temperature optimum of the three AMDases. Enzyme activities of the AMDases were measured in a temperature range from 4 to 50°C. **(B)** Optimal pH conditions were measured in a range from pH 4 to pH 9 using citric acid (CA), sodium phosphate (NaPB), potassium phosphate (KPB) and Tris buffer (Tris). **(C)** Solvent stabilities of the enzymes were measured in ethanol (EtOH), methanol (MetOH), 2-propanol and DMSO at a final concentration of 10%. **(D)** Influence of putative cofactors and salts on AMDase activity were measured in 1 mmol of sodium phosphate buffer (NaPP), pyridoxal 5′-phosphate (PP), nicotinamide adenine dinucleotide (NAD), ATP, thiamine diphosphate (TD), ZnCl_2_, NaCl and MgCl_2_. All tests were carried out using 10 mM phenylmalonic acid as substrate and its conversion was measured by HPLC.

For further investigations the enzymes AmdA, AmdV, and AmdP were chosen. AmdA and AmdV originated from *Variovorax* sp. HH01 resp. *Variovorax* sp. HH02. The *Variovorax* strains are isolates from one soil sample and showed the highest activity on indicator medium plates. Furthermore, special interest lies in them as no other enzymes from this genus have been characterized so far. AmdP has been found by data base search as a candidate to prove the applicability and reliability of the searching criteria. The corresponding gene originated from *Polymorphum gilvum* SL003B-26A1, a strain which was originally isolated from a crude oil-polluted saline soil and was able to grow at temperatures up to 50°C (Nie et al., 2012). This makes AmdP a promising candidate for putative industrial applications. The enzymatic properties of the newly identified AmdA, AmdP, and AmdV were assayed using recombinant purified protein. A turnover of 10 mM of the substrate phenylmalonic acid was measured by HPLC. Concerning the temperature optimum, all three enzymes had their optimal activity in a comparable mesophilic range between 30 and 37°C (**Figure 6A**). AmdV showed maximal activity at 30°C and possessed 44 ± 3% activity at 10°C. Above 30°C, however, activity rapidly decreased with 6 ± 2% remaining at 50°C. The highest activity of AmdA from *Variovorax* sp. HH01 could be detected at 34°C. At 10°C, the activity decreased to 33% and above 40°C, activity decreased rapidly until almost no enzyme activity could be measure above 50°C. The highest conversion of the substrate by AmdP could be measured at 37°C. Below this temperature, the activity of this enzyme was lower compared to AmdA and AmdV, at 10°C even 2.6-fold lower than the activity of AmdV. Above 37°C, AmdP showed a better activity compared to the other two AMDases with 4.8-fold more substrate conversion at 45°C than AmdV.

The optimal pH conditions of the enzymes were assayed using phenylmalonic acid substrate solutions. All three enzymes are pH-dependent and showed the highest activity at a neutral to slightly acidic pH, mostly with Tris and sodium phosphate buffer (**Figure 6B**). AmdA showed its highest activity at pH 6.0, and AmdV as well as AmdP at a pH of 7.0 (Tris buffer). Below pH 4.0, no enzymatic activity could be detected, whereas at a pH of 8.0 and 9.0, 85% residual activity could be measured for AmdA and at pH 9.0, more than 65% activity remained in case of AmdV and AmdP. The preference for neutral or slightly basic conditions agrees well with that of the well-characterized AMDase from *B. bronchiseptica* (Miyamoto and Ohta, 1992a).

The solvent stabilities of the three AMDases were measured in 10% solutions (v/v) of ethanol, methanol, isopropanol, and DMSO diluted in 10 mM Tris buffer pH 7.0. AmdA, AmdV, and AmdP were similarly susceptible to the negative influences of the solvents (**Figure 6C**). Ethanol and isopropanol decreased the enzyme activities of the AMDases to less than 10% of the controls without solvent. With methanol, the substrate turnover was also strongly reduced to approx. 15% (AmdA 13 ± 5%, AmdV 15 ± 2%, and AmdP 16 ± 2%). DMSO reduced the enzymatic activity of AmdA and AmdV only to approx. half of the control (48 ± 4 and 50 ± 4%, respectively) and AmdP still possessed 82 ± 7% activity in this solvent.

Concerning the influence of salts and possible cofactors, none of the tested effectors sodium phosphate, pyridoxal phosphate, NAD, ATP, thiamine diphosphate, zinc chloride, sodium chloride, and magnesium chloride (all 1 mM in final concentration) showed a beneficial effect on the enzymatic activity of AmdA, AmdV, and AmdP, proving that the enzymes function cofactor-independently (**Figure 6D**). Magnesium and especially zinc chloride even reduced enzyme activity significantly (MgCl_2_: 81 ± 1% residual activity for AmdA and 83 ± 5% for AmdV. ZnCl_2_: 74 ± 9% for AmdV, 38 ± 5% for AmdA and 8 ± 4% for AmdP compared to the effector-less control).

**FIGURE 7:**
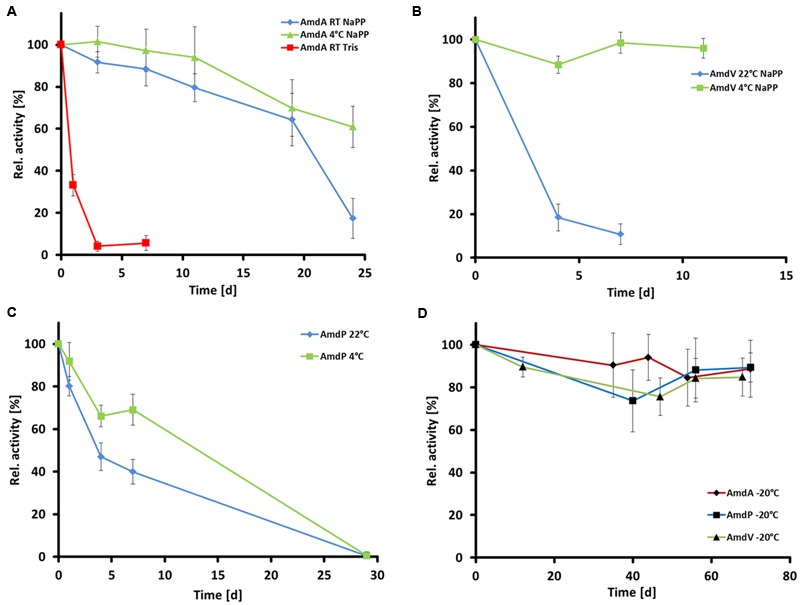
**Temperature stability of the three recombinant and purifed AMDases AmdA (*Variovorax* sp. HH01), AmdV (*Variovorax* sp. HH02), and AmdP (*Polymorphum gilvum* SL003B-26A1).** Measurements are triplicates with three technical repeats. **(A)** Temperature stability of AmdA in 50 mM Tris buffer (Tris) and 50 mM sodium phosphate buffer (NaPP) at 4°C and room temperature (22°C, RT) within 30 days. **(B)** Temperature stability of AmdV in 50 mM sodium phosphate buffer (NaPP) at 4°C and room temperature (22°C, RT) within 11 days. **(C)** Temperature stability of AmdP in 50 mM sodium phosphate buffer (NaPP) at 4°C and room temperature (22°C, RT) within 30 days. **(D)** Temperature stability at -20°C of all three enzymes in 50 mM sodium phosphate buffer.

The thermal stabilities and optimal storage conditions of the three AMDases were assayed at 22, 4, and -20°C in 50 mM Tris buffer pH 7.0 and 50 mM sodium phosphate buffer pH 7.0, respectively, by measuring the enzymes’ residual activities. In case of all three enzymes, storage in sodium phosphate buffer seemed to be beneficial compared to Tris buffer (**Figure 7A**). The residual activity of AmdA (*Variovorax* sp. HH01) after 7 days at 22°C in Tris buffer comprised only 6 ± 4% while in sodium phosphate buffer, 64 ± 10% activity were still measurable after 19 days at the same temperature. The best storage conditions for AmdA were at 4°C in sodium phosphate buffer with 61 ± 12% residual activity after 24 days. AmdP from *P. gilvum* SL003B-26A1 was stable for more than 7 days with 40 ± 6% residual activity after incubation at 22°C and with 69 ± 7% at 4°C (**Figure 7C**). Although AmdV (*Variovorax* sp. HH02) lost most of its activity after storage at 22°C for 7 days (11 ± 5% residual activity), it was highly stable at 4°C and retained 96 ± 5% of its initial activity after 11 days (**Figure 7B**). As shown in **Figure 7D**, for a long-term storage of the three enzymes, it is recommendable to choose sodium phosphate buffer (50 mM and pH 7.0) and -20°C as the AMDases keep more than 85% of their initial activity after at least 70 days (AmdA 89 ± 13%; AmdV 85 ± 9%, and AmdP 89 ± 7%).

### Selectivity of AmdA, AmdV, and AmdP on Derivatives of 2-Arylpropionic Acids

**FIGURE 8:**
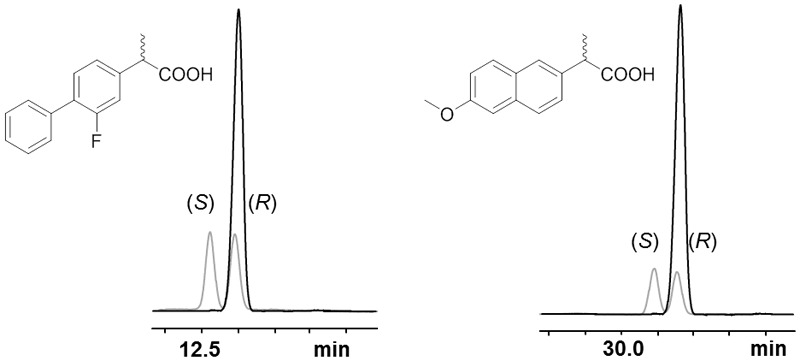
**Selectivity of AmdA, AmdV, and AmdP on derivatives of 2-arylpropionic acids.** Selected GC-chromatograms of (*R*)-flurbiprofen **(left)** and (*R*)-naproxen **(right)** after AMDase-catalyzed decarboxylation (here using AmdA) of the corresponding aryl malonic acids. Racemic arylpropionates are showαn in gray. The measurement was done using a FS-Hydrodex-β-6-TBDM column from Macherey-Nagel with an isothermic temperature of 160°C for naproxen and 170°C for flurbiprofen.

The selectivity of all three AMDases was analyzed with aryl malonic acid derivates of the arylpropionic acids naproxen and flurbiprofen. All enzymes showed a very high (*R*)-selectivity for both substrates as the obtained *ee*-values were greater than 99% indicating a huge excess of the (*R*)-enantiomer (**Figures 1A** and **8**). In the case of flurbiprofen, spontaneous decarboxylation during the preparation of the aryl malonic starting material limited the *ee*. In the synthesis of naproxen, all three AMDases showed *(R)*-preference and a very high enantioselectivity (>99% *ee*). In the case of flurbiprofen, the malonic acid is of limited stability. During the synthesis of the starting material, a small amount of racemic flurbiprofen (<4%) is formed that cannot be separated from the flurbiprofen malonate. As a consequence of this contamination, the enantiomeric excess of flurbiprofen is somewhat decreased. This effect is known and can be circumvented by the optimization of the reaction conditions. Nevertheless, the selectivity of the three AMDases in the synthesis of flurbiprofen can be assumed to be very high. Terao et al. (2003) showed an *ee* of only 92% for (*R*)-flurbiprofen using the AMDase from *B. bronchiseptica*. This effect is known and can be circumvented by the optimization of the reaction conditions (Gaßmeyer et al., 2016). In the synthesis of naproxen, all three AMDases showed (*R*)-preference and a very high enantioselectivity (>99% ee).

## Discussion

Within this work, we have developed a novel search algorithm, allowing the identification of AMDase gene candidates from genomes and metagenomes. Altogether we were able to identify 58 novel possible enzyme candidates. A first classification of AMDases due to their protein sequence similarities and occurrence of conserved homologous genes in the sequence environment of *amd* enabled the formation of eight enzyme clusters within this work (**Figure 5**). Moreover, it should be noted that a large number of proteins have been automatically annotated as AMDases. However, as these proteins lack essential catalytic features like the hydrophobic binding pocket, an activity toward arylmalonates is thus highly unlikely. We confirmed this by cloning and testing the gene WP_013776825 from *Acidianus hospitalis*. Common features in the *amd* flanking sequences are the occurrence of genes encoding TRAP, ABC, and TTTs. Furthermore striking was the prevalence of a gene coding for MR/MLE, which was present in 61% of the available sequences. Five clusters comprise strains with genes coding for TTTs next to their *amd* gene (**Figure 5B**). Alpha-proteobacterial strains of the TTT clusters contain genes encoding all three subunits of TTTs. In contrast, all of the beta-proteobacterial representatives just carry “orphan” genes encoding periplasmic receptor proteins (**Figure 5A**). Genes coding for TctC are known to be overrepresented in *Beta-proteobacteria* (Antoine et al., 2003). Only a small amount is part of polycistronic operons. The majority of these so-called *bug* (***B****ordetella* **u**ptake **g**ene) genes are “orphan” genes whose products most probably operate as extracytoplasmic substrate receptors (Antoine et al., 2003). There are suggestions that different receptor proteins interact with the same TctAB subunits (Antoine et al., 2003). Alternatively, the receptor protein BctC from *B. pertussis* appears to be involved in signal transduction by interacting with the periplasmic domain of the sensor protein BctE (Antoine et al., 2005). The high number of TTT cluster’s representatives might indicate an evolutionary origin of AMDases within these strains or at least beneficial conditions that have been favored by these clusters. TRAP transporters are tripartite, ATP independent periplasmic transporters that have been found in bacteria and archea and enable the uptake of C_4_-carboxlyates and further organic anions, e.g., lactate, glutamate, gluconate, ectoine, or benzoate derivatives by using an electrochemical gradient (Forward et al., 1997; Kelly and Thomas, 2001; Fischer et al., 2010; Mulligan et al., 2011). The uptake of 4-chlorobenzoate in *Comamonas* sp. DJ-12 is carried out by the TRAP transporter FcbT1-T3 (Chae and Zylstra, 2006). The corresponding genes are part of an inducible operon together with a gene encoding an enzyme that catalyzes the conversion of 4-chlorobenzoate to 4-hydroxybenzoate. An analogous correlation between genes of TRAP transporters and AMDases is conceivable. ABC transporter genes adjacent to *amd* genes are annotated as putative peptide/nickel transporters (Navarro et al., 1993). Genes downstream of *amd* encode two ATP binding domains. Genes upstream of *amd* encoding a permease subunit and a substrate binding protein, are separated by an ORF annotated as putative oxidoreductase. The insertion of these two genes makes the functionality of the ABC transporters questionable although single genes are conserved. Because of the frequent occurrence of genes coding for various transporters next to the amd genes, it is likely that they are involved in substrate uptake.

**FIGURE 9:**
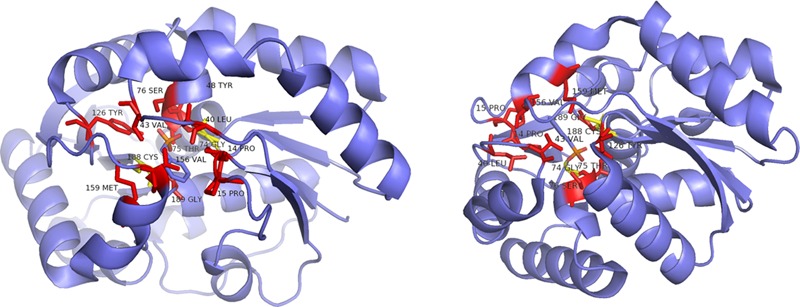
**Ribbon representation of the *B. bronchiseptica* enzyme Gly74/Cys188 (structure derived from PDB 3IP8) from two different perspectives.** Red: parts of the hydrophobic binding pocket and the dioxyanion hole; yellow: catalytic residues determining the stereoisomery of the product, both shown in a stick representation (Okrasa et al., 2009).

The enzyme properties of the newly characterized AMDases from *Variovorax* sp. and *P. gilvum* fit well into the overall picture of known AMDases with an optimum pH from 5.5 to 8.5 and the highest substrate conversion at temperatures from 30 to 45°C (Miyamoto and Ohta, 1992a; Miyamoto et al., 2007, 2008; Okrasa et al., 2008). So far, all known wildtype AMDases have a strict selectivity for the formation of the (*R*)-enantiomer. This (*R*)-selectivity is based in the position of the catalytic Cys at position 188 in the active site (**Figure 9**). By moving this residue to the opposite side to position 74, the enantioselectivity of AMDase could be switched to an (*S*)-preference (Terao et al., 2007). None of the identified 58 putative AMDases, however, has a Cys in position 74, indicating that the natural enantiopreference of AMDase is strictly for the (*R*)-enantiomer. The selectivity seems to be highly conserved through all known AMDases. As the natural substrate of AMDase remains to be identified, and it is not even known if it is prochiral, the highly conserved enantiopreference remains a puzzling feature of the decarboxylases.

All *amd* genes have a high GC content that is mostly higher than the average GC content of the corresponding genome (**Supplementary Figure S1**). Genomes with high GC content are characteristic for soil organisms with complex metabolic abilities (Foerstner et al., 2005). The restriction to *Proteobacteria* could indicate a recent evolution within this group. The isolated occurrence of AMDases in most of the known genera with only one representative per species or strain implies that AMDases provide a slight competitive advantage for their carriers and might be lost in most bacteria.

The recent findings on AMDases described in this publication will significantly extend the knowledge of these enzymes and provide promising candidates for the production of enantiopure flurbiprofen. The eight novel enzyme clusters give new insights to possible reactions preceding decarboxylation (e.g., substrate uptake) and enzymatic reactions taking place subsequently to this step. Besides, the clustering shows the restriction of this conserved *Bordetella* prototype to some classes of *Proteobacteria*. Together with the newly established AMDase-specific search algorithm the screening for novel AMDases in data bases will be improved and will enable an effective use of the continuously growing sequence data for a reliable prediction of AMDase activity for the very first time.

The screening method is particularly useful for enzymes with a known structure and reaction mechanism. The generated HMM enables a specific search for AMDases of the *Bordetella* prototyp and is beneficial for time-saving screenings of massive sequence data like metagenomic samples. Accordingly, the chance of finding biocatalysts with suitable properties can be increased dramatically. Furthermore, it is an appropriate method to find organisms as well as habitats where the desired enzymes occur and are more or less abundant. This allows a first insight into possible physiological and ecological functions and consequently also their natural substrate.

## Author Contributions

Conceived and designed the experiments: JM, JC, RK, and WS. Performed the experiments: JM, SaG, SiG, and FB. Analyzed the data: JM, JC, SaG, RK, and WS. Contributed reagents/materials/analysis tools: RK and WS. Contributed to the writing of the manuscript: JM, JC, SaG, SiG, RK, and WS.

## Conflict of Interest Statement

The authors declare that the research was conducted in the absence of any commercial or financial relationships that could be construed as a potential conflict of interest.

## Footnotes

1 http://www.ncbi.nlm.nih.gov/

2 https://img.jgi.doe.gov/cgi-bin/er/main.cgi

3 http://blast.ncbi.nlm.nih.gov/Blast.cgi

4 www.rcsb.org

5 http://www.mbio.ncsu.edu/bioedit/bioedit.html

6 http://staden.sourceforge.net/

7 http://doua.prabi.fr/software/seaview

8 www.skylign.org

9 http://pfam.xfam.org/

## Supplementary Material

FIGURE S1 **GC content of *amd* genes in comparison to the average genomic GC content.** The information on the GC contents was retrieved from the genomes listed in Supplementary Table S1 that have been deposited in the IMG database.

TABLE S1 **List of putative *amd* genes found in this work**.
