# Supplementary figures and images for: Sequence-Based Screening for Rare Enzymes: New Insights into the World of AMDases Reveal a Conserved Motif and 58 Novel Enzymes Clustering in Eight Distinct Families
Source: Front Microbiol. 2016 Aug 25;7:1332. doi: 10.3389/fmicb.2016.01332 (PMC4996985; doi:10.3389/fmicb.2016.01332)

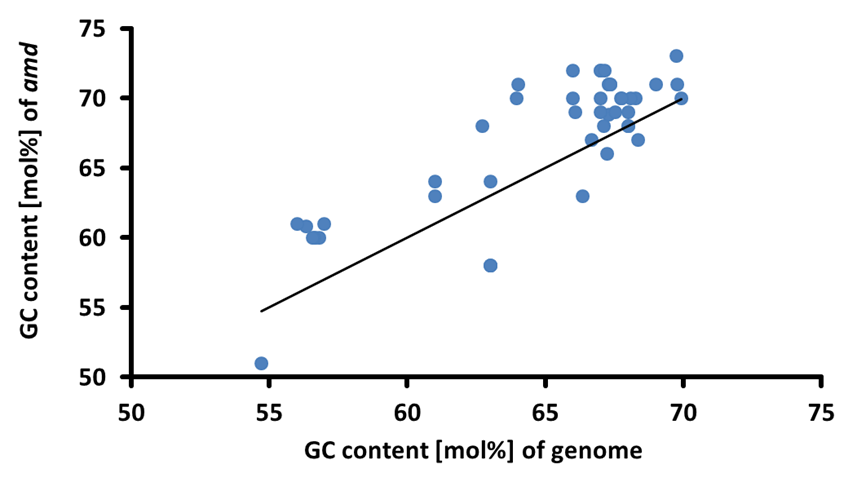

Supplement: FIGURE S1 — GC content of amd genes in comparison to the average genomic GC content. The information on the GC contents was retrieved from the genomes listed in Supplementary Table S1 that have been deposited in the IMG database. [file Image_1.TIF]
