# Supplementary material for: Sequence-Based Screening for Rare Enzymes: New Insights into the World of AMDases Reveal a Conserved Motif and 58 Novel Enzymes Clustering in Eight Distinct Families
Source: Front Microbiol. 2016 Aug 25;7:1332. doi: 10.3389/fmicb.2016.01332 (PMC4996985; doi:10.3389/fmicb.2016.01332)
Supplement: TABLE S1 — List of putative amd genes found in this work. [file Data_Sheet_1.DOCX]

**Tab. S1: List of putative *amd* genes found in this work.**

| Cluster | Locus Tag (IMG/NCBI) |  | Organism | Isolation source |
| --- | --- | --- | --- | --- |
| ABC-I | G489DRAFT_03382 |  | *Cucumibacter marinus*DSM 18995 | Costal seawater |
|  | Ga0077207_102085 |  | *Devosia* limi DSM 17137 | Activated sludge, nitrifying inoculum |
|  | Ga0057540_03866 |  | *Devosia* sp. 17-2-E-8 | Soil |
|  | Ga0055148_11612 |  | *Devosia* sp. DBB001 | Not mentioned |
|  | NA2_11794 |  | *Nitratireductor pacificus* pht-3B | Sediment from the Pacific Ocean |
| TRAP-I | PA01_RS00445 |  | *Azoarcus* sp. PA01 | Wastewater treatment plant |
|  | Ga0070015_11733 |  | *Halomonas* sp. DSM 26667 | Not mentioned |
|  | RO22_RS01255 |  | *Halomonas* sp. KHS3 | Seawater |
|  | SL003B_3169 |  | *Polymorphum gilvum* SL003B-26A1 | Crude oil contaminated saline soil |
|  | H610DRAFT_00387 |  | *Pseudomonas azotifigens* DSM 17556 | Hyperthermal compost material |
|  | Ga0055015_102642 |  | *Roseivivax isoporae* LMG 25204 | Institute for ozeanography |
| TRAP-II | TR61_RS04585 |  | *Aestuariivita boseongensis* | Tidal flat sediment |
|  | AIOL_RS13050 |  | *Candidatus* Rhodobacter lobularis | Endosymbiont of *Oscarella lobularis* (Homoscleromorph sponge) |
|  | MesJ18_0055.00000160 |  | *Mesorhizobium* sp. J18 | Genome sequencing of lignin-degrading bacterial isolates |
|  | NA2_08029  NA2_11794 |  | *Nitratireductor pacificus* pht-3B  *Nitratireductor pacificus* pht-3B | Pyrene-degrading consortium of an enriched sediment from the Pacific Ocean |
|  | Z946_RS0115425 |  | *Sulfitobacter noctilucicola* (NB-77) | Sea sparkle bloom region |
| TTT-I | n.a. |  | *Achromobacter* sp. HH01 | Soil enriched with phenylmalonate as sole carbon source |
|  | WP_055891218.1 |  | *Achromobacter* sp. (Strain 2789STDY5608625) | Cystic fibrosis sputum of a patient (carriage) |
|  | n.a. |  | *Achromobacter* sp. KU1311 | Soil enriched with phenylmalonate as sole carbon source |
|  | MC81_10270 |  | *Achromobacter xylosoxidans* (strain FDAARGOS_88) | Endotracheal aspirate |
|  | Ga0077226_10648 |  | *Bordetella bronchiseptica* KU1201 | Soil enriched with phenylmalonate as sole carbon source |
|  | n.a. |  | *Enterobacter cloacae* KU1313 | Soil enriched with phenylmalonate as sole carbon source |
| TTT-II | QWA_04274 |  | *Alcaligenes faecalis faecalis* NCIB 8687 | Isolated from arsenical cattle-dipping fluids, Arsenite oxidizer |
|  | Ga0077136_102325 |  | *Alcaligenes faecalis* MOR02 | Not mentioned |
|  | G456DRAFT_02398 |  | *Alcaligenes faecalis phenolicus* DSM 16503 | Wastewater bioprocessor |
|  | Ga0056709_02091 |  | *Alcaligenes faecalis* subsp. *faecalis* NBRC 13111 | Not mentioned |
|  | Ga0077189_103128 |  | *Alcaligenes faecalis* ZD02 | Agricultural |
|  | N879_11820 |  | *Alcaligenes* sp. EGD-AK7 | Not mentioned |
|  | C660_17402 |  | *Alcaligenes* sp. HPC1271 | Activated biomass of an effluent treatment plant treating industrial wastewater |
| TTT-III | D827DRAFT_03400 |  | *Pseudacidovorax intermedius* NH-1 | Marine |
|  | ASG30_RS00930 |  | *Ramlibacter sp. Leaf400* | Leaf of *Arabidopsis thaliana* |
|  | G369DRAFT_02382 |  | *Variovorax paradoxus* 110B | Rhizosphere and endosphere of *Arabidopsis thaliana* |
|  | D461DRAFT_00741 |  | *Variovorax paradoxus* 4MFCol3.1 | Rhizosphere and endosphere of *Arabidopsis thaliana* |
|  | VAPA_1c13830 |  | *Variovorax paradoxus* B4 | Industrial wastewater |
|  | Ga0078369_101825 |  | *Variovorax paradoxus* MEDvA23 | Microbial strains from phyllosphere of *Arabidopsis thaliana* |
|  | Vapar_1310 |  | *Variovorax paradoxus* S110 | Interior of a potato plant |
|  | APR52_RS20595 (APR52_20590) |  | *Variovorax paradoxus* strain H061 | Biofilm in a CDC reactor with chloraminated drinking water |
|  | APR49_RS09215 |  | *Variovorax paradoxus* strain H084 | Biofilm in a CDC reactor with chloraminated drinking water |
|  | APR50_RS10335 |  | *Variovorax paradoxus* strain H090 | Biofilm in a CDC reactor with chloraminated drinking water |
|  | APR51_RS07820 |  | *Variovorax paradoxus* strain H095 | Biofilm in a CDC reactor with chloraminated drinking water |
|  | APR47_RS26665 |  | *Variovorax paradoxus* strain H108 | Biofilm in a CDC reactor with chloraminated drinking water |
|  | APR48_RS17760 |  | *Variovorax paradoxus* strain H112 | Biofilm in a CDC reactor with chloraminated drinking water |
|  | VPARA_RS10210 (VPARA_20570) |  | *Variovorax paradoxus* strain TBEA6 | Contaminated soil |
|  | L980_05797 |  | *Variovorax* *paradoxus* ZNC0006 | Bacteria isolated from zebrafish intestine |
|  | Ga0059266_5714 |  | *Variovorax* sp. 770b2 | Forest soil (loam) communities across North America |
|  | Ga0059267_4952 |  | *Variovorax* sp. EL159 | Forest soil (loam) communities across North America |
|  | Ga0099533_11097 |  | *Variovorax* sp. HH01 | Soil Botanical Garden Duisburg, Germany |
|  | Ga0099534_11097 |  | *Variovorax* sp. HH02 | Soil Botanical Garden Duisburg, Germany |
|  | Ga0058956_05108 |  | *Variovorax* sp. NFACC26 | Bacterial root endophytes of switchgrass |
|  | Ga0058957_03218 |  | *Variovorax* sp. NFACC27 | Bacterial root endophytes of switchgrass |
|  | Ga0058958_05218 |  | *Variovorax* sp. NFACC28 | Bacterial root endophytes of switchgrass |
|  | Ga0058959_05229 |  | *Variovorax* sp. NFACC29 | Bacterial root endophytes of switchgrass |
|  | Ga0070142_102175 |  | *Variovorax* sp. OK605 | Populus root microbiome, host associated, endophyte, rhizoplane |
|  | Ga0102127_101398 |  | *Variovorax* sp. Root473 | Roots of *Arabidopsis thaliana*, green house |
| TTT-IV | AmiJ15_0043.00000150 |  | *Aminobacter* sp. J15 | Lignin-degrading bacterial isolates |
|  | AmiJ41DRAFT_00035970 |  | *Aminobacter* sp. J41 | Lignin-degrading bacterial isolates |
|  | AmiJ44_0001.0000158 |  | *Aminobacter* sp. J44 | Lignin-degrading bacterial isolates |
|  | BO39DRAFT_04377 |  | *Roseomonas aerilata* DSM 19363 | Air |
|  | Ga0070241_11928 |  | *Tranquillimonas alkanivorans* DSM 19547 | Not mentioned |
| TTT-V | C507DRAFT_01657 |  | *Amorphus coralli* DSM 19760 | Coral mucus, host *Pleuractis granulosa* |
|  | Meso_3050 |  | *Chelativorans* sp. BNC1 | Mixed-culture enriched from sewage using the chelating agent EDTA |
|  | BO59DRAFT_03721 |  | *Ponticoccus litoralis* DSM 18986 | Costal seawater |
|  | OCH239_14795 |  | *Roseivivax halodurans* JCM 10272 | Not mentioned |

Adapted nucleotide sequence of *Polymorphum gilvum* *amdP* gene (IMG locus tag SL003B-26A1):

>ATGACCTCTCGTCCGGGTGATGGACGTCCCCTGGTAGGCCTGATTGTGCCACCAGCCACAGGTTTGGTCCCTCCGGAACCTCCAGCGCTGTATGGCGATGCGCTGCGCTTTGCAGCCCGTGGCTTAGCCTTAGCCACCATGACGCGCGATGGGTACGACGACGTGATCGATCGCGTTGAAGCGGCAGCGCGCGCTTTGGCGGCGGAAGGTGCAGCCGCCGTTGCACTGATGGGCACCAGTCTGTCGTTCTATCGGGGTGCTGCGTTCAATGACGCCCTTGTTGAGCGCATGGCGTCTGCGACTGGCCTGCCGGTGACGACGATGTCCTGCGCAGTTGTCGAGGCCCTGCGCGCAGTTGGCGCTCGTCGCTTAGCGGTGGCGACAGCCTACGTCGATGAGGTGAACGATCGCCTGACGGCGTTTCTGCTCCATCACGGCTTTGAGGTGCTCGGACTGGATTCGCTGCAAATCAGTGCGGTAGGTGATGTCCTTGCGATTGGCGATGACGACCTGATTGGGCTCGGAACCCGCGCGTTCGTCGCTGCTCCGGAAGCCGACGCACTTCTGGTGAGCTGTGGTGGGTTACAGACCCTGTCCGTTACCTTGCCCTTGGAAGATCGTCTGGGCGTGCCGGTGATCAGCTCAGCCGTAGCCGGCGCATGGGCTGCAGCGCGGCTGATTGGCCATGGTGGTGAAGCACCGGGGTATGGTCGTCTGCTCGAAACTGCTCCGAAAAGCGAACTCGAG
